# Unmasking Tuberculosis: A Case of Pericardial Effusion in a Young Adult With Recurrent Pneumonia

**DOI:** 10.7759/cureus.89361

**Published:** 2025-08-04

**Authors:** Majid Elalami, Anthony Semaan, Marco Valladres, Kim Nguyen, Jhoette Dumlao

**Affiliations:** 1 Family Medicine, Chino Valley Medical Center, Chino, USA

**Keywords:** acute pericardial effusion, multiviral pneumonia, productive cough, sob - shortness of breath, tubercolosis

## Abstract

This case presents a 25-year-old Indian male with no significant past medical history presenting to the emergency department (ED) due to two weeks of productive cough with pleuritic chest pain. The patient presented one week earlier to the ED; however, he left against medical advice and was given a 5-day course of Azithromycin 250 mg that minimally improved his symptoms. He returned to the ED shortly after completing the antibiotics and was admitted for further evaluation. He was diagnosed with multifocal pneumonia and started on intravenous antibiotics, then discharged two days later on oral outpatient therapy. One month later, the patient returned to the ED with similar symptoms of worsening productive cough and pleuritic chest pain. CT chest findings revealed a left-sided pleural effusion and large pericardial effusion, which later prompted microbiological testing that confirmed a Mycobacterium tuberculosis infection. A pericardial window was indicated due to tamponade physiology. Although the patient did not present with classic constitutional symptoms of tuberculosis, this case shows the importance of keeping TB high in the differential list among those with recurrent pneumonia and unexplained pleural and pericardial effusions, especially in patients with recent immigration or insidious risk factors, despite how rare pathologies such as pericardial TB can be. Early correct diagnosis and appropriate diagnostic workup, including imaging and microbiological studies, should be ordered to prevent delay in treatment and reduce morbidity.

## Introduction

Tuberculosis is one of the leading causes of mortality and morbidity worldwide [1]. In 2024 alone, an estimated 10.8 million people fell ill with TB, with over 1.25 million deaths reported globally, underscoring its persistent public health impact, especially in low and middle-income countries [2]. Mycobacterium tuberculosis, the causative agent of tuberculosis, is primarily transmitted through the respiratory system and affects the lungs, leading to pulmonary TB; however, the rest of the body can be affected. Although pulmonary TB is the most common form, extrapulmonary manifestations of TB can occur, involving the pericardium and pleura. Pericardial effusion (PE) is the accumulation of excess fluid in the sac surrounding the heart, which, when undiagnosed, can cause compression of the heart, leading to cardiac tamponade. Tuberculosis pericarditis (TBP) is rare and can account for 1-2% of all TB infections [3]. TBP is the most frequent cause of large pericardial effusions in developing countries and represents the leading cause of constrictive pericarditis in adults [4]. This condition is associated with significant morbidity and a high risk of mortality. When a young, healthy 25-year-old male presents with an acute-onset cough and pleuritic chest pain, tuberculosis is not often high on the differential list, unlike common etiologies such as pneumonia and bronchitis. In this report, we introduce a 25-year-old Indian male originally treated for multifocal pneumonia, later diagnosed with a pericardial effusion with the potential of developing life-threatening tuberculosis pericarditis.

## Case presentation

A 25-year-old male with no significant past medical history presented to the ED on 1/29/25 at 1800 due to worsening cough. On arrival to the ED, the patient endorsed having 15 days of productive cough with yellow sputum associated with pleuritic chest pain. He denied shortness of breath, chills, fever, night sweats, and weight changes. The patient visited India two years ago and denied any known exposure to TB. The patient reported coming to the ED five days prior on 1/24/25 with similar symptoms of persistent cough and was discharged with a five-day course of azithromycin, which he completed and reported minimal improvement in symptoms. The patient denies a history of smoking, alcohol, and drug use. The patient was febrile, tachycardic, and tachypneic in the ED. His vital signs were as follows: blood pressure (BP) 107/65, temperature 100.4 degrees F, heart rate (HR) 108, respiratory rate (RR) 23, and oxygen saturation (SpO2) 97% on room air. The physical exam noted rhonchi and decreased breath sounds on the back of the left lung lobes.

**Figure 1:**
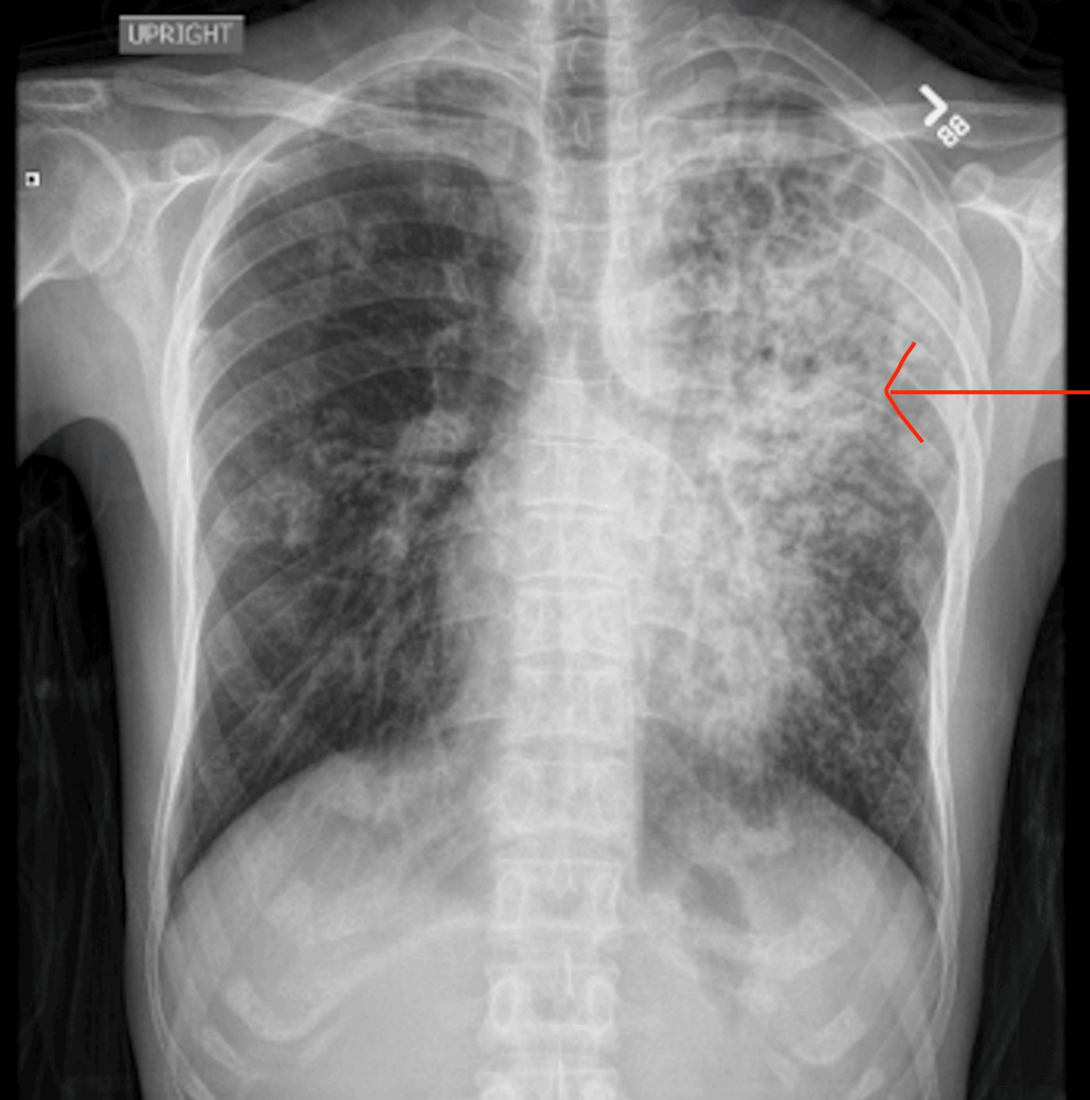
Chest X-ray showing multifocal pneumonia The red arrow is pointing to areas of consolidation and interstitial markings, consistent with multifocal pneumonia.

In the emergency department, treatment consisted of Zosyn with 1 L of normal saline. Labs, including complete blood count, metabolic panel, and chest X-ray, were ordered (Table 1). Chest X-ray on 1/29/25 (Figure 1) showed findings consistent with multifocal pneumonia, greater on the left compared to the right.

**Table 1:** Complete blood count on 2/19/2025 MCV: mean corpuscular volume; MCHC: mean corpuscular hemoglobin concentration; RDW: red cell distribution width; MPV: mean platelet volume

Parameter	Value	Reference Range	Interpretation
WBC (×10³/µL)	10.8 ↑	4.0–11.0	Slightly elevated
RBC (×10⁶/µL)	4.00 ↓	4.5–5.9 (M), 4.1–5.1 (F)	Low
Hemoglobin (g/dL)	10.9 ↓	13.5–17.5 (M), 12.0–15.5 (F)	Anemia
Hematocrit (%)	32.7 ↓	38.8–50.0 (M), 34.9–44.5 (F)	Anemia
MCV (fL)	81.9	80–100	Normal
MCH (pg)	27.2 ↓	27–33	Borderline low
MCHC (g/dL)	33.2	32–36	Normal
RDW (%)	15.8 ↑	11.5–14.5	Elevated
Platelets (×10³/µL)	348	150–450	Normal
MPV (fL)	7.0 ↓	7.5–11.5	Low
Neutrophils (%)	82.2 ↑	40–75	High
Lymphocytes (%)	5.7 ↓	20–45	Low
Monocytes (%)	11.3	2–10	Slightly elevated
Eosinophils (%)	0.1 ↓	1–4	Low
Basophils (%)	0.7	0–1	Normal
Abs Neutrophils (×10³/µL)	8.9	1.5–8.0	Elevated
Abs Lymphocytes (×10³/µL)	0.6 ↓	1.0–3.0	Low
Abs Monocytes	1.2	0.2–0.8	Elevated

The patient was admitted to the medical-surgical/acute level of care for pneumonia. The top differential included sepsis secondary to multifocal pneumonia. Blood culture, urinalysis, urine culture, and sputum cultures were ordered. The patient received Zosyn every eight hours and Doxycycline twice a day (BID) due to tachycardia with IV fluids at 100 ml/hour. Other differentials included microcytic anemia secondary to iron deficiency, transaminitis, new-onset prediabetes, hyponatremia, hypocalcemia, and severe protein-calorie malnutrition (Table 2).

**Table 2:** Chemistry profile on 2/19/2025 BUN: blood urea nitrogen; AST: aspartate aminotransferase; ALT: alanine transaminase; A/G: albumin/globulin

Parameter	Value	Reference Range	Interpretation
Sodium (mmol/L)	126 ↓	135–145	Hyponatremia
Potassium (mmol/L)	4.3	3.5–5.0	Normal
Chloride (mmol/L)	90	98–106	Low
CO₂ (Bicarb) (mmol/L)	30.5 ↑	22–29	High
Anion Gap (mmol/L)	5.5	8–16	Low
BUN (mg/dL)	9	7–20	Normal
Creatinine (mg/dL)	0.6	0.6–1.2	Normal
BUN/Creatinine Ratio	15	10–20	Normal
Glucose (mg/dL)	117 ↑	70–99 (fasting)	Mildly elevated
AST (U/L)	71 ↑	10–40	Elevated
ALT (U/L)	72 ↑	7–56	Elevated
Alkaline Phosphatase (U/L)	110	44–147	Normal
Total Protein (g/dL)	7.4	6.0–8.3	Normal
Albumin (g/dL)	2.1 ↓	3.4–5.4	Low
Globulin (g/dL)	5.3	2.0–3.5 (calc)	High
A/G Ratio	0.4 ↓	1.0–2.5	Low
Total Bilirubin (mg/dL)	0.7	0.1–1.2	Normal

On 1/31/25, the patient was stable for discharge, as he showed clinical improvement throughout his hospitalization with normalizing white blood count, and he was saturating well on room air with stable vitals. The patient was deemed stable for discharge with Augmentin and Doxycycline BID for seven days. The patient was also recommended to follow up with his primary care provider for transaminitis, as the patient was found to have an AST of 141 and an ALT of 153 during his hospital stay.

**Figure 2:**
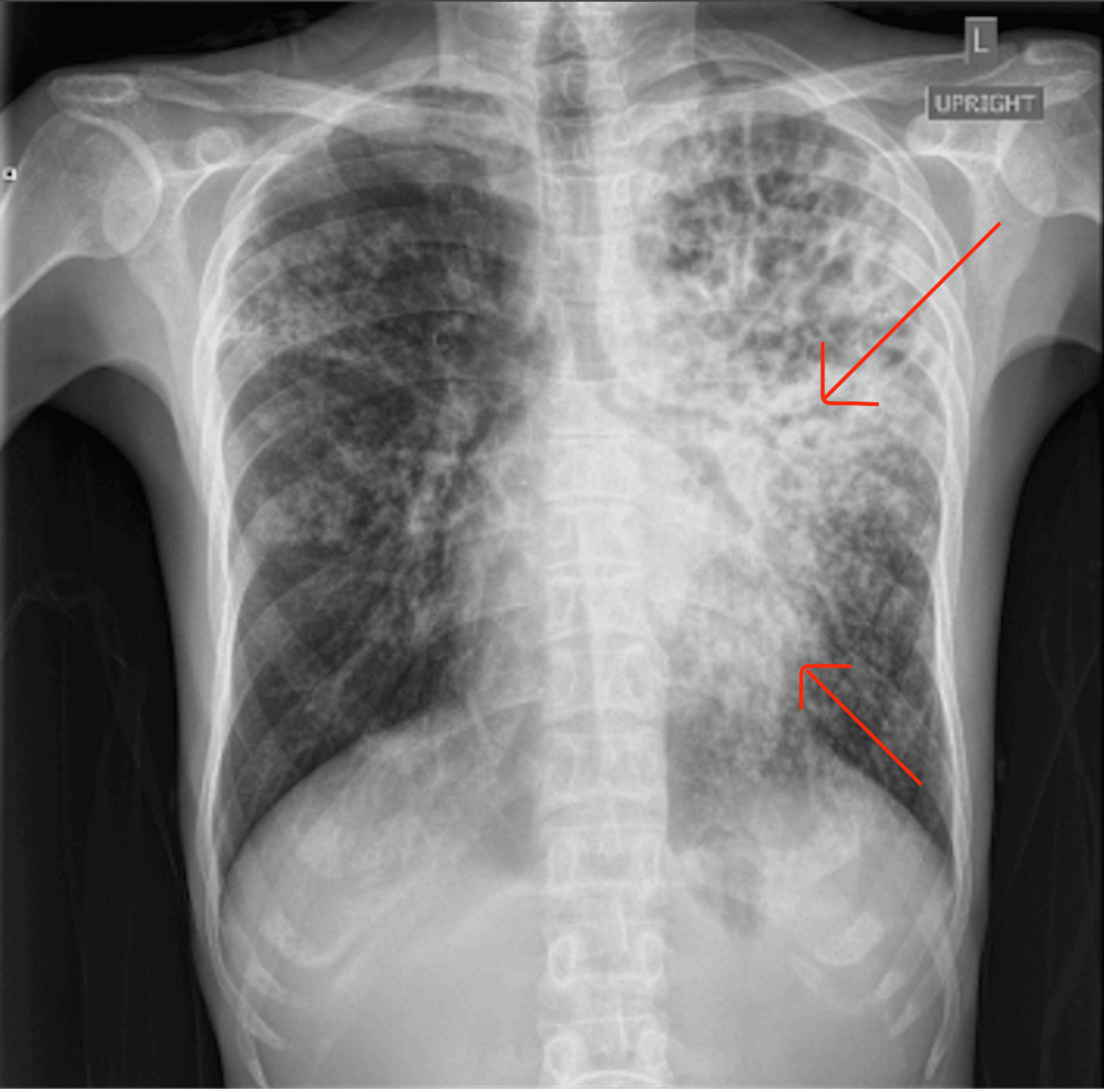
X-ray chest showing interstitial and patchy airspace disease The red arrow indicates bilateral interstitial and patchy airspace disease, mostly unchanged from the prior X-ray.

On 02/19/25, the patient was readmitted to the hospital, as he presented with similar symptoms of a productive cough with associated pleuritic chest pain. He reports that he had been compliant with his antibiotic regimen. He denied fever, chills, and night sweats. He had been saturating well on room air, but stated that the cough had been bothering him. Vital signs on admission were as follows: BP 101/65, Temp 98.0, HR 118, RR 18, and SpO2 sat 97 % on room air. The physical exam included diffuse crackles in all lung fields bilaterally, worse on the left side. X-ray findings on 2/19/25 (Figure 2) demonstrated interstitial and patchy airspace disease in both lungs, greater on the left, likely multifocal pneumonia. The patient was admitted for community-acquired pneumonia with failed outpatient treatment. Upon admission, IV Vancomycin and Cefepime 1g BID were started. CT (catscan) chest with and without contrast, sputum culture, quantiferon, HIV rapid, urine tox, blood cultures, and urine legionella and histoplasmosis were ordered. 

**Figure 3:**
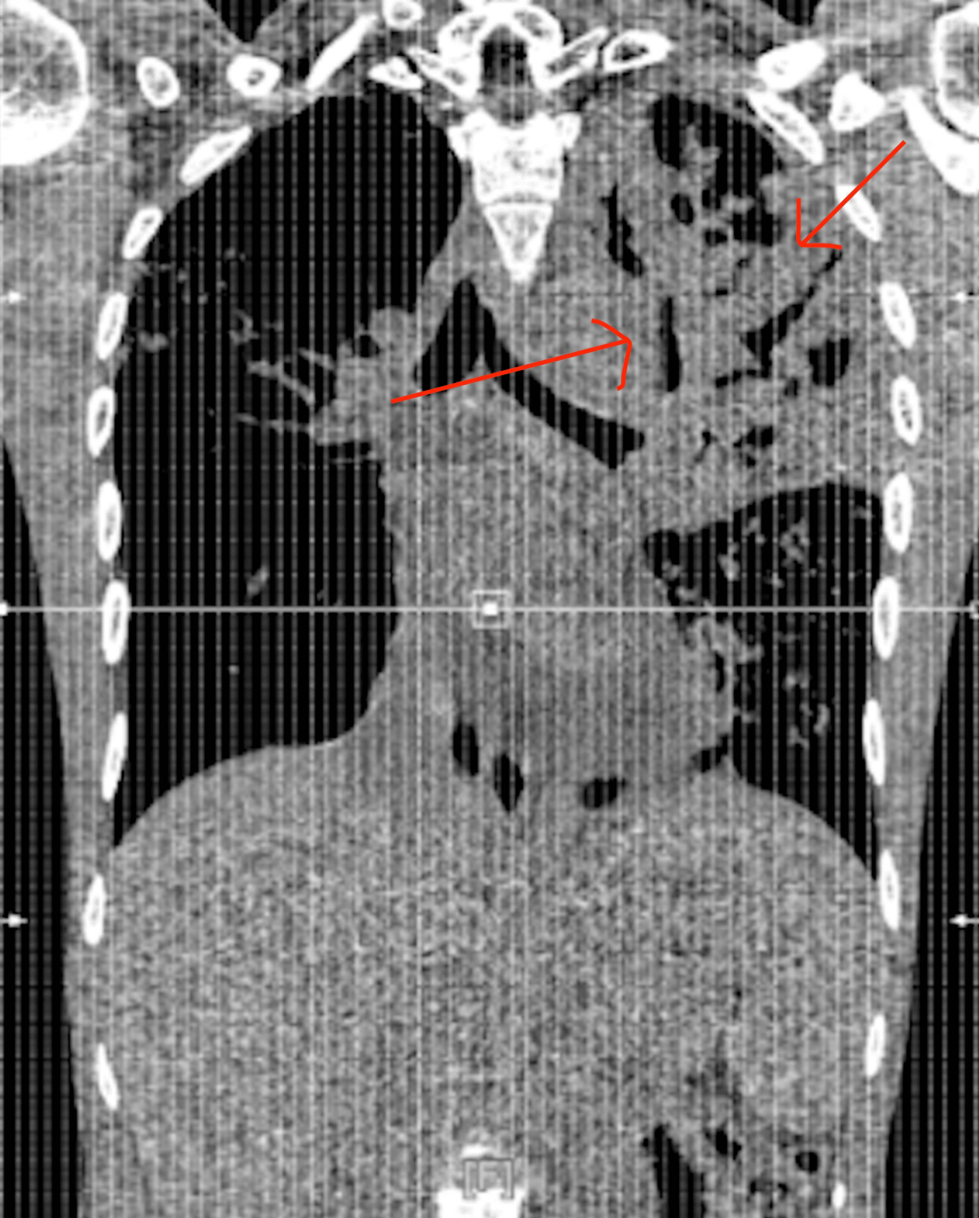
Catscan chest without contrast with left upper lobe cavitary lesions and infiltrates The red arrow is pointing to cavitary lesions in the left upper lobe of the lung, findings on the CT scan concerning for multifocal pneumonia, which was also seen in multiple X-rays.

CT chest without contrast on 2/19/25 (Figure 3) had pulmonary findings concerning for atypical and multifocal pneumonia, moderate to severe pericardial effusion, and trace left-sided pleural effusion. Cardiology was consulted for pericardial effusion. Echocardiogram was performed and showed the left ventricle was normal in size with an estimated ejection fraction of 55-60%; however, there was a large circumferential pericardial effusion with echocardiographic evidence of tamponade physiology.

**Figure 4:**
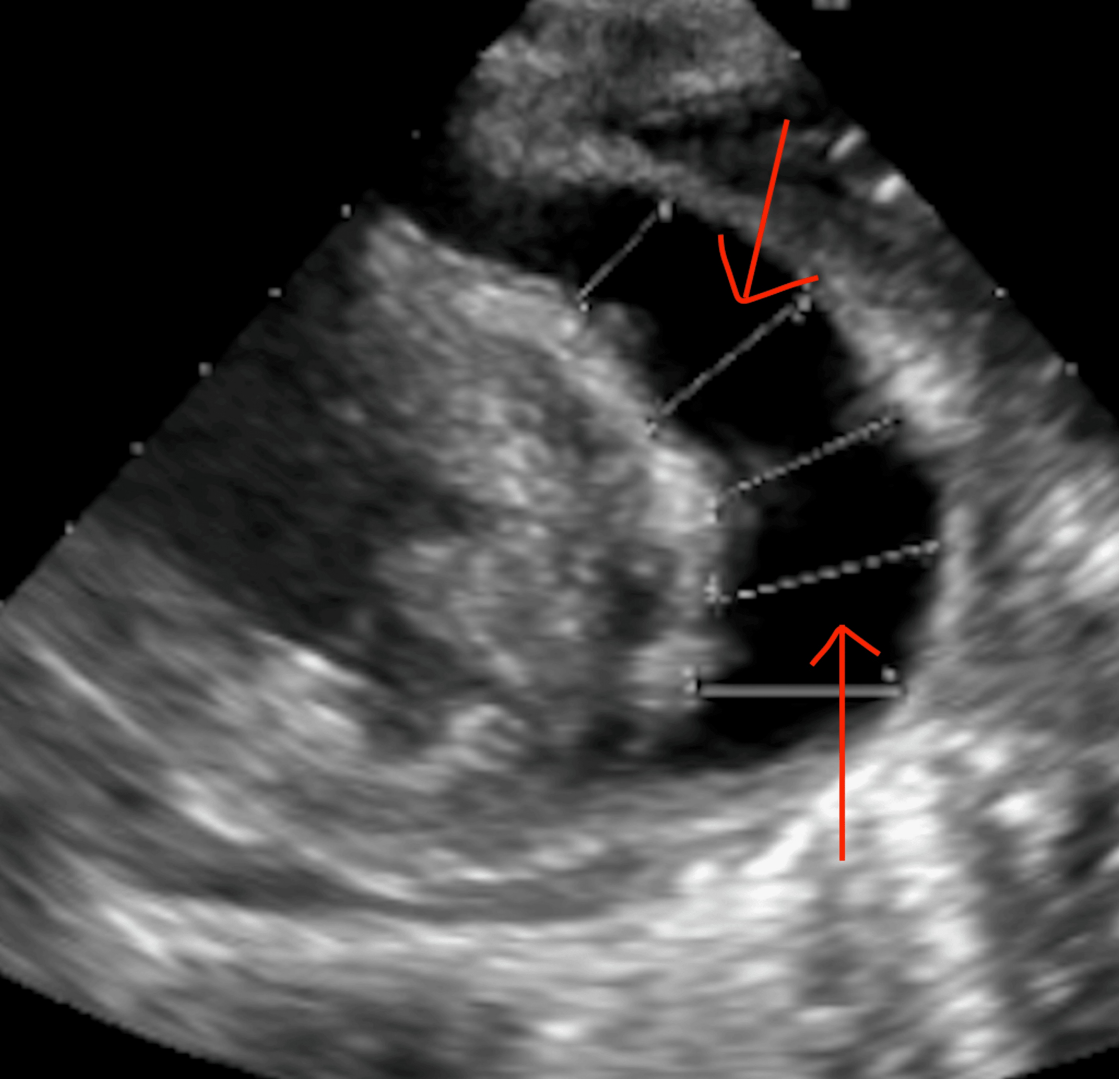
Echocardiogram with large pericardial effusion The red arrow is pointing to a large (anechoic) space between the heart and pericardium, consistent with a pericardial effusion.

Sputum TB testing returned strongly positive. CT chest was reviewed and showed infiltrates, particularly severe in the left upper lobe, with small probable cavitary lesions in the left upper lobe. Echo (Figure 4) confirmed a large pericardial effusion with signs of tamponade. A pericardial window was indicated. The patient appeared to have TB pneumonia, likely with superimposed bacterial pneumonia.

The pericardial window was performed on 2/23/25, and 1 L of watery, clear pericardial fluid was drained. Complete evacuation of fluid was confirmed on transesophageal echo. There was no exudate on the heart or pericardium as would be expected with TB pericarditis. Acid-fast bacilli (AFB) smear was noted to be positive on 2/26/25, following the procedure, which was the diagnostic method used for diagnosis. TB (tuberculosis) treatment was notified to Public Health, and the patient was recommended to start RIPE (Rifampin, Isoniazid, Pyrazinamide, and Ethambutol) therapy.

## Discussion

This case illustrated a presentation of tuberculosis with extrapulmonary involvement in a young immunocompetent patient without classical symptoms of TB. It is difficult to diagnose tuberculosis (TB), especially when it presents in extrapulmonary ways with pericardial and pleural involvement. The patient initially presented with community-acquired pneumonia symptoms, and the lack of fever, night sweats, and weight loss lowered the clinical suspicion for TB. One important lesson from this case is acknowledging TB as a differential diagnosis for a patient with persistent respiratory symptoms, especially in individuals who have epidemiologic risk factors, including recent travel to endemic countries.

A critical aspect of this case was the evolving radiologic picture, which, in retrospect, offered early clues suggestive of tuberculosis. The initial chest X-ray showed multifocal pneumonia, predominantly left-sided, a finding that should have raised concern given the patient’s epidemiologic background. On readmission, imaging progressed to demonstrate bilateral interstitial and patchy airspace disease with cavitary lesions in the left upper lobe on CT scan. Cavitation is a hallmark feature of reactivation, particularly in the apical or posterior segments. Additionally, the presence of both pleural and pericardial effusions on CT should have broadened the differential to include TB early in the course. This case highlights how underrecognition of TB-specific imaging patterns, especially in high-risk populations, can contribute to delayed diagnosis. Integrating radiologic findings with clinical and epidemiologic risk factors is essential for the timely identification of extrapulmonary TB and the appropriate escalation of care.

Pleural TB is one of the most common forms of extrapulmonary TB and presents with an exudative pleural effusion, which can be the sole manifestation of TB [5]. In this case, our patient presented with a unilateral pleural effusion and large pericardial effusion on CT, which prompted AFB testing, ultimately confirming the presence of TB. The diagnostic modality of choice for a pericardial effusion is an echocardiogram, which showed signs of tamponade physiology in our patient, prompting treatment with a pericardial window [6].

In developing countries, tuberculosis and cancer are the predominant causes of pericardial effusions, with TB being the most frequent [7]. Extrapulmonary tuberculosis (EPTB) can affect nearly any organ system but is frequently overlooked or misdiagnosed due to its nonspecific clinical features and limited access to diagnostic tissue [8]. The pleura and lymph nodes are the most commonly involved sites, followed by the bones, joints, gastrointestinal tract, peritoneum, kidneys, genitourinary system, and meninges [9].

Indications for TB testing include but are not limited to persistent respiratory symptoms, particularly a cough lasting longer than three weeks, abnormal chest findings, such as pleural effusions, and the presence of epidemiologic risk factors for latent TB infection [10]. The epidemiologic risk factors include prior residence or travel to a TB-endemic area. Despite meeting these criteria, our patient was not initially evaluated for TB, largely due to the absence of classic constitutional symptoms and a clinical presentation suggestive of community-acquired pneumonia. This delay underscores a key pitfall in TB diagnosis, which is overreliance on classic symptoms of TB and failure to account for epidemiologic context. Earlier recognition of the patient's risk factors and radiographic findings may have expedited appropriate TB testing and intervention, potentially preventing the progression to a life-threatening pericardial effusion with tamponade physiology.

The patient was initially discharged with symptomatic treatment; however, he returned one month later with worsening pleuritic chest pain. On readmission, echocardiography revealed a new small pericardial effusion, raising concern for progression to extrapulmonary tuberculosis. Early comprehensive evaluation is warranted for patients with unresolved pneumonias or atypical presentations, which include chest CT, echocardiography, and microbiologic studies. This case illustrates the importance of timely TB screening and contact tracing in suspected cases and low-suspicion cases, as early detection and successful treatment can reduce the spread of TB and the development of resistant strains.

## Conclusions

This case highlights a rare but critical presentation of extrapulmonary tuberculosis manifesting as pericardial effusion with tamponade physiology in a young, otherwise healthy patient. Despite the absence of classical constitutional symptoms, the patient's recurrent respiratory complaints, persistent imaging abnormalities, and epidemiologic risk factors were key in identifying tuberculosis as the underlying etiology. Early recognition of ordering a chest CT and echocardiography into the diagnostic workup led to timely intervention with a pericardial window, preventing potentially fatal outcomes. This case demonstrates the importance of maintaining a high index of suspicion for TB in patients with unexplained effusions to avoid diagnostic delays and reduce morbidity associated with extrapulmonary TB manifestations.
